# The Amorphous Urate Deposit

**Published:** 1888-04

**Authors:** 


### EDITORIAL.

### THE AMORPHOUS URATE DEPOSIT.

The above forms a portion of the title of a valuable paper contributed to *The Medical Chronicle* (March, 1888) by Sir William Roberts. The author first abstracts the paper of Dr. Bence Jones (*Journal of the Chemical Society*, 1862). He gives an unqualified assent to the propositions enunciated in that paper, the grounds for which he has carefully investigated. He adopts the nomenclature of Dr. Jones, and also his formula; considering the amorphous urates to consist of quadurates of potassium, sodium, and ammonium having the general formula:

$$\left\{\begin{matrix}\left.\begin{matrix}H\\H\end{matrix}\right\}\breve{u}\\ \left.\begin{matrix}M\\H\end{matrix}\right\}\breve{u}\end{matrix}\right\}$$

These amorphous urates, after being washed with alcohol on a filter, or better still, separated from the fluid portions of the urine by allowing them to fall upon a sheet of blotting-paper, are found to readily decompose on the addition of water. A minute portion placed under a microscope will show the gradual formation of uric-acid crystals when water is added. Assuming, then, that the uric-acid in the urine exists normally in the urine in the form of quadurates, the question is asked, why are they deposited in the form of amorphous urates, instead of being broken up into bi-urates and uric acid?

The author, in experimenting with normal urine upon the purified deposit, found that the inhibitory power was comparative and not absolute. The time occupied in this change varied inversely as to its concentration. It was further found that, if urine was dialysed with an equal volume of water, it lost for the most part its power of preventing the precipitation of uric acid from the amorphous deposit. This fact led to the inference that the crystalloids of the urine were the chief factors in preventing this change. Solutions of these various crystalloids were prepared and their effect in preventing the change noted. Uric acid was deposited from solutions of urea almost as promptly as in simple water. The chlorides and sulphates, in the proportion of 1 per centum and upwards, exerted considerable delay, but did not approach the normal urine in its power of preventing the decomposition of quadurates. The most pronounced effects were obtained from phosphate of potassium, a solution of 0.2 per centum acting as perfectly as normal urine, while those as dilute as one part in 2,000, sensibly delayed the process as compared with pure water.

The importance of this discovery cannot be overestimated, and must in a great measure modify our views regarding the cause and pathology of calculous disease and gouty concretions—as Dr. Roberts says that the soundness of the inference is substantiated by Mr. Plowright, who conclusively shows that abundance of salt in the food hindered the formation of calculus. This he erroneously attributed to the increased solubility of uric acid in saline solutions; whereas, it is really due to the power salines have of preventing the decomposition of the quadurates.

It is also apparent that the hypothetical "acid fermentations" described by Scherer, in which the aid of a special fungus is invoked to liberate the uric acid in neutral sodium urate, must be abandoned, at least, in so far as the first portion of the process is concerned, and likewise the theories of Brücke & Röhmann.

